# A Study on the Incidence and Impact of Dysglycemia in Non-diabetic Sepsis Patients

**DOI:** 10.7759/cureus.66546

**Published:** 2024-08-09

**Authors:** Jerin Varghese, Naveen Mohan, Indresh Kumar, Gireesh Kumar, Sreekrishnan Trikkur, Sabarish Nair, Bharath Prasad, Manna M Theresa, Midhun Viswanath

**Affiliations:** 1 Emergency Medicine, Amrita School of Medicine, Kochi, IND; 2 Emergency Medicine, Amrita School of Medicine, Faridabad, IND

**Keywords:** apache ii, sepsis, non diabetic, hyperglycemia, hypoglycemia

## Abstract

Context

Dysglycemia is common in severe sepsis and is associated with a poor prognosis. There is a limited amount of research on stress-induced dysglycemia in non-diabetic sepsis patients.

Aim

This study aims to estimate the incidence of dysglycemia among non-diabetic patients presenting with sepsis at the Emergency Department and to determine its correlation with gender, age, APACHE II (Acute Physiology and Chronic Health Evaluation) scores, diagnosis, and duration of hospital stay.

Materials and methods

The study was conducted at a medical college hospital in Kochi from January 1, 2023, to December 31, 2023. A minimum sample size of 77 was derived after a pilot study, with a 95% confidence interval and 10% allowable error. A total of 100 non-diabetic sepsis patients meeting the inclusion and exclusion criteria were analyzed with regard to gender, age, diagnosis, glycemic status (hypo/hyper/normoglycemic), APACHE II scores, and hospital stay duration. Statistical analysis was performed using IBM SPSS Statistics for Windows, Version 20 (Released 2011; IBM Corp., Armonk, New York) software. Categorical variables were expressed as frequency and percentage. Continuous variables were presented as mean ± SD (standard deviation) and median (Q1-Q3). To test the statistical significance of the association between the presence of various factors (gender, age, diagnosis) and dysglycemia, the chi-square test was used. To test the statistical significance of the difference in the mean age and APACHE II score values with dysglycemia, an independent sample t-test was used. To test the statistical significance of the difference in the median hospital stay with dysglycemia, the Whitney U test was used. Data were represented as mean ± SD, and a p-value of <0.05 was considered to be statistically significant.

Results

The incidence of dysglycemia in the inclusion group was 49% (hypoglycemia in 16% and hyperglycemia in 33% of cases), and it increased with age (p=0.002). The majority of the dysglycemic patients fell into the age group >40 years. Dysglycemia was 54.8% in pneumonia and 66.7% in gastrointestinal sepsis ( p=0.138). Dysglycemia increased with an increase in APACHE II scores (p=0.017). The median hospital stay was almost the same in both normoglycemics and dysglycemics.

Conclusion

Dysglycemia is a frequent complication in non-diabetic patients with sepsis. It increased with age and APACHE II score, but it does not prolong the duration of hospital stay, nor is it associated with the diagnosis.

## Introduction

Background

Hyperglycemia and hypoglycemia are frequently observed in the intensive care unit (ICU), particularly among critically ill patients with diabetes. Hyperglycemia is more prevalent than hypoglycemia, and if the blood glucose is uncontrolled, it often leads to fluid and electrolyte disturbances, increased infection rates, and, ultimately, mortality [1]. There is a scarcity of research on dysglycemia in non-diabetic patients who arrive at the emergency room with sepsis. Therefore, there is an increasing research focus in this area.

Inflammatory response and stress (resulting from sepsis and critical illness) are found to induce significant alteration in glucose homeostasis and thereby result in dysglycemic states. Blood glucose levels in sepsis are maintained by a complex interaction between the proinflammatory mediators (TNF-α, IL-1, and IL-6) and the counter-regulatory hormones (glucagon, cortisol, catecholamine, and growth hormone). Hyperglycemia occurs when there is an increase in mechanisms such as hepatic gluconeogenesis, hepatic glycogenolysis, lipolysis, and muscle glycolysis, combined with increased insulin resistance. It typically manifests during periods of high stress, such as in cases of sepsis. Hyperglycemia may also be caused by exogenous steroids, dextrose infusions, parenteral and enteral nutrition, and inflammatory cytokines. Hyperglycemia in elderly individuals is also attributed to coexisting morbidities and decreased physiological and immunologic responses. The liver can also produce glucose from lactate via the Cori cycle, especially during stress. This can also result in hyperglycemia [2-4].

Hyperglycemia will ultimately lead to volume depletion, hypoperfusion, electrolyte imbalances, and acid-base disturbances. At the tissue level, hyperglycemia leads to reduced nitric oxide, superoxide generation, endothelial dysfunction, platelet activation, immune dysregulation, and mitochondrial injury. Consequently, this leads to impaired phagocytic activity of neutrophils, increased cytokine production, and multiorgan failure in severe sepsis patients [5].

Hypoglycemia, although less prevalent, can occur due to a variety of factors, whether they are endogenous or exogenous. Endogenous reasons include sepsis, hyperinsulinism (insulinoma, non-insulinoma, pancreatogenous hypoglycemia, and autoimmune hypoglycemia), functional β-cell disorders, or the insulin autoimmune syndrome, reduced gastrointestinal absorption, underlying unknown liver disease, viral hepatitis, massive hepatic destruction, impaired gluconeogenesis, hepatic glycogen store depletion, glycogen storage diseases, those with renal failure, decreased renal gluconeogenesis, decreased renal insulin clearance, resolution of uremia post-renal replacement therapy, and severe cardiac disease (pathology less understood). Exogenous reasons include reduced calorie intake, metformin consumers (such as for polycystic ovarian disease), decreased metabolism of hypoglycemia-causing drugs, and excessive alcohol consumption.

Certain drugs, malicious administration of insulin, herbal supplements (fenugreek), alcohol, underlying eating disorders, liver disease, hypothyroidism, post-stomach resection, or end-stage renal disease are the causes attributed to fasting hypoglycemia. Hyperinsulinism, prediabetes, and digestive tract surgeries are attributed to reactive hypoglycemia. Acute pancreatitis, obesity, drugs causing catecholamine depletion, immunosuppressants (tacrolimus, cyclosporine), and corticosteroids are other factors enhancing hypoglycemia. Additionally, patients with sepsis who were fasting or those with acute or chronic liver disease during initial presentation have a high predilection for hypoglycemia. However, the mechanism of hypoglycemia in sepsis remains unknown.

Regarding the long-term consequences of dysglycemia, Leonidau et al. (2008) conducted a study on the clinical and lab characteristics of 265 patients with severe sepsis and baseline hypoglycemia. They concluded that stress-induced hypoglycemia is related to a more severe disease and poorer prognosis [6]. Another study by Umpierezz et al. stated that hyperglycemia in non-diabetics required intensive care evaluation and that it increased mortality in non-diabetics compared to diabetics [7]. Van den Burghe et al., in a non-blinded RCT (randomized controlled trial) on 1548 patients, concluded that strict blood sugar control in the ICU reduced the mortality rate as well as the duration of ICU stay, ventilator support, blood transfusion, renal impairment, and bloodstream infections [8]. In a study done by Miller et al. (1980), hypoglycemia with overwhelming sepsis resulted in an overall mortality of 67% [9].

Objective

The primary objective of our study is to estimate the incidence of dysglycemia among non-diabetic patients presenting with sepsis at the emergency department. The secondary objective is to determine its correlation with gender, age, APACHE II (Acute Physiology and Chronic Health Evaluation) scores, diagnosis, and duration of hospital stay.

## Materials and methods

Study design

A prospective observational study was conducted on 100 non-diabetic sepsis patients. The study was approved by the hospital ethics committee.

Setting

The study was conducted from January 1, 2023, to December 31, 2023, at the Emergency Medicine Department and Emergency Intensive Care Unit (ICU) of an Indian Medical College Hospital with an annual emergency department patient load of approximately 50,000.

Participants

A convenient sampling strategy was used. A total of 185 non-diabetic participants with sepsis as per the verbal history were screened. Unfortunately, 85 of them turned out to be diabetic as they had elevated HbA1c reports (>6.5) and were excluded. Hence, only 100 participants were included in the final assessment, as our study group consisted exclusively of non-diabetics. Those with initial GRBS >140 mg/dL were labeled as hyperglycemia, those with initial GRBS <70 mg/dL were labeled as hypoglycemia, and others were labeled as normoglycemia.

Variables

The quantitative variables include age, initial GRBS (serum glucose), HbA1c, temperature, mean arterial pressure, pH, heart rate, respiratory rate, serum sodium, serum potassium, creatinine, hematocrit, WBC count, Glasgow Coma Scale (GCS), FiO_2_, and duration of hospital stay (in days). The qualitative variables consist of medical records department (MRD) number, gender, comorbidities, diagnosis, history of severe organ failure/immunocompromise, and glycemic status (normoglycemic/hypoglycemic/hyperglycemic). Derived variables include the APACHE II score (derived by an online MDCALC calculator).

Data sources/measurement

A total of 100 patients meeting the inclusion and exclusion criteria (as mentioned below) were studied. The inclusion criteria include (1) non-diabetic patients (HbA1c between 4 and 6.5) and (2) patients with two or more of the following objective parameters: oral temperature >38 °C or <36 °C, respiratory rate >20 breaths per minute, heart rate >90 beats per minute, WBC count >12000/µL or <4000/µL with suspected or proven infection. The exclusion criteria include patients with pancreatitis, burns, trauma, pulmonary embolism, myocardial infarction, anaphylaxis, drug overdose, those on steroids, drugs interfering with glycemic control (e.g., quinolones), and post-gastrointestinal resection surgeries.

Patients were classified based on their age, gender, diagnosis, glycemic status, and APACHE II scores and followed up to determine the duration of hospital stay (in days). The demographics are presented in Table 1.

**Table 1:** Demographics

Variables	Frequency
Age (mean±SD)	52.1±17.5
Gender (males/females)	(59/41)
Gastrointestinal sepsis	15
Urosepsis	12
Pneumonia	31
Glycemia status (normal/hyperglycemia/hypoglycemia)	(51/33/16)
Hospital stay (mean±SD)	11±11.6
APACHE II score (mean±SD)	16.0±7.3

Operational definitions

The operational definitions of the study are presented in Table 2.

**Table 2:** Operational definitions

Term	Definition
Sepsis	Life-threatening organ dysfunction caused by a dysregulated host response to infection (Surviving Sepsis Campaign Guidelines 2021)
Normoglycemia	Serum glucose: 70-140 mg/dL
Hypoglycemia	<70 mg/dL
Hyperglycemia	>140 mg/dL
HbA1c	normal 4%-6.5%

Bias

The quantitative data were collected by emergency medical technicians and staff nurses on duty, while the qualitative data were collected by the emergency physicians on duty.

Study size

A minimum sample size of 77 non-diabetics was derived (with 95% confidence and 10% allowable error) from a pilot study. For this study, we took 185 cases by convenient sampling during the study period, and 100 were included after the exclusion.

Quantitative variables

The quantitative variables include age (in years), GRBS (in mg/dL), HbA1c (in %), temperature(in °C), mean arterial pressure (in mmHg), pH, heart rate (in beats per minute), respiratory rate (in rate per minute), serum sodium (mEq/L), serum potassium (mEq/L), serum creatinine (mg/dL), hematocrit (%), WBC count (/µL), GCS, FiO_2_ (%), and duration of hospital stay (in days).

Statistical analysis

Continuous variables were expressed as mean ± SD. Statistical analysis was done using the IBM SPSS Statistics for Windows, Version 20 (Released 2011; IBM Corp., Armonk, New York) software. To test the statistical significance of the association between the presence of various factors (gender, age, diagnosis) with dysglycemia, a chi-square test was used. To test the statistical significance of the difference in the mean of age and APACHE II score values with dysglycemia, an independent samples t-test was used. To test the statistical significance of the difference in the median hospital stay with dysglycemia, the Mann-Whitney U test was used. Data were represented as mean ± SD, and a p-value <0.05 was considered to be statistically significant.

## Results

Participants

**Figure 1:**
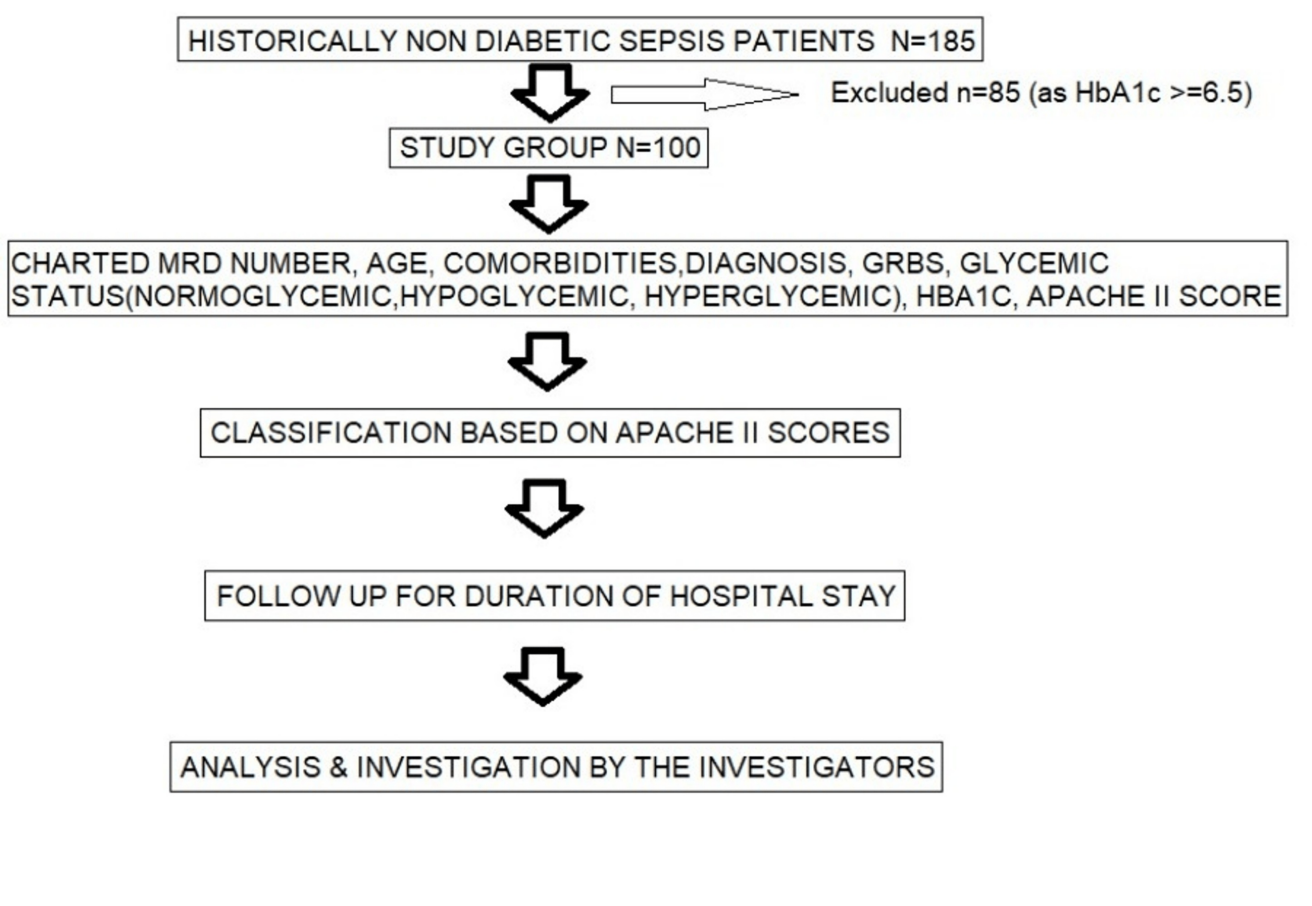
Flow diagram

A total of 100 non-diabetic sepsis patients were subjected to the study, as mentioned in the flow diagram (Figure 1).

Descriptive data

The total incidence of dysglycemia among the study group was 49%: hypoglycemia in 16% and hyperglycemia in 33% of the cases. A total of 31 males had dysglycemia (52.5%), whereas only 18 females had dysglycemia (43.9%). This was found to be statistically insignificant (Table 3).

**Table 3:** Incidence of dysglycemia and gender distribution (n=100)

Category	Male	Female	Total
Normoglycemics	28 (28%)	23 (23%)	51 (51%)
Hyperglycemics	21 (21%)	12 (12%)	33 (33%)
Hypoglycemics	10 (10%)	6 (6%)	16 (16%)
Total	59 (59%)	41 (41%)	100 (100%)
Chi-square value=0.728; p=0.695			

The majority of the dysglycemic patients fall in the greater than 40-year age group. The dysglycemic percentage was higher (67.6%) in the more than 60-year age group. This shows that the incidence of dysglycemia increases with age, and this is also found to be statistically significant (Table 4).

**Table 4:** Age vs. dysglycemia distribution

Age (Years)	Dysglycemics	Normoglycemics	Total	% Dysglycemia
21 to 40	4	17	21 (21%)	19%
41 to 60	22	23	45 (45%)	48.9%
>60	23	11	34 (34%)	67.6%
Total	49	51	100 (100%)	49%
Chi-square value=12.27; p=0.002 (significant)				

The mean APACHE II score of dysglycemia patients was 17.1±8.298, and it was 14.3±5.7 for those without dysglycemia. The mean was higher in dysglycemic patients. Dysglycemia increased with an increase in APACHE II scores. A p-value of 0.017 and a t-value of -2.42 was obtained, indicating a clear difference (Table 5).

**Table 5:** APACHE II score vs. dysglycemia

APACHE II Score	No. of Patients	Dysglycemics	% Dysglycemia
0 to 5	5	4	80
6 to 10	20	7	35
11 to 15	26	9	34.6
16 to 20	20	8	40
21 to 25	16	10	62.5
26 to 30	11	9	81.8
31 to 35	2	2	100
p-value=0.017; t-value= -2.42			

The most common diagnosis in non-diabetic sepsis was pneumonia (31%) followed by gastrointestinal sepsis (15%). Dysglycemia was 54.8% in pneumonia and 66.7% in gastrointestinal sepsis, with a p=0.138 and chi-square value of 2.20, which is not statistically significant (Table 6).

**Table 6:** Diagnosis in non-diabetic sepsis vs. dysglycemia

Diagnosis	Dysglycemics	Normoglycemics	Total	% Dysglycemia	P-value	Chi-Square Value
Gastrointestinal sepsis	10	5	15	66.7	0.138	2.20
Urosepsis	4	8	12	33.3	0.358	1.34
Pneumonia	17	14	31	54.8	0.434	0.613
Other	18	24	42	42.8		

The median hospital stay for dysglycemic patients was nine days with an interquartile range of 6-14.5 (Q1-Q3). The median hospital stay for normoglycemic septic patients was eight days with an interquartile range of 7-12 (Q1-Q3). There is no significant difference in the median hospital stay concerning glycemic status (p-value of 0.507 and z-value of -0.664).

## Discussion

Key results

The incidence of dysglycemia in sepsis was 49% (hypoglycemia in 16% and hyperglycemia in 33% cases) and increased with age (p=0.002). The majority of the patients fall into the >40-year age group, and 67.6% of the patients in the >60-year age group had dysglycemia. Dysglycemia was 54.8% in pneumonia and 66.7% in gastrointestinal sepsis (p=0.138). Dysglycemia increased with an increase in APACHE II scores (p=0.017). The median hospital stay was almost the same for both normoglycemics and dysglycemics, and no significant correlation was obtained in this case.

Limitations

Firstly, there is likely some selection bias present, particularly in the data collection phase for qualitative characteristics, as it was collected by the emergency physicians on duty. To mitigate this, they were provided with previous knowledge of the study as well as clear instructions regarding the parameters that would be evaluated. Secondly, the study looks only at the initial GRBS of the patients. Serial serum glucose monitoring will be required for days together in the respective ICUs to obtain a clearer picture. Thirdly, non-diabetic patients who may have already received a dose of corticosteroids from a previous hospital for various reasons but not documented in their medical records may unknowingly intrude into our study group. This could potentially alter the serum glucose level, hence affecting the findings of the study.

Interpretation

It was observed that the incidence of hyperglycemia was higher than that of hypoglycemia among non-diabetic sepsis patients presenting to the emergency department, but it is statistically insignificant. Several prior studies have also demonstrated a higher incidence of hyperglycemia compared to hypoglycemia in non-diabetic patients with sepsis. Kushimoto et al. found that in a cohort of patients with severe sepsis, only 6% had hypoglycemia on admission, while 27% presented with hyperglycemia [10].

Similarly, a review by Nugent et al. [11] highlighted that hyperglycemia is a more frequent complication in sepsis, even in individuals without a history of diabetes. Another multicenter study by Sathananthan et al. (2020) found an initial hyperglycemia rate of 27.3% in non-diabetic septic patients [12]. Another subanalysis of the data derived from the FORECAST study reported the rate of initial hyperglycemia as 15.3% and the rate of early hypoglycemic episodes as 9.3%, in non-diabetic sepsis patients without glycemic control [13]. The disparity in our study compared to previous studies may be attributed primarily to a selection bias and secondarily to a convenient sampling strategy. Hence, we suggest a random sampling strategy with complete blinding of the data collectors.

Secondly, our study revealed that the elderly are more prone to dysglycemia during sepsis. This may be attributed to probable etiologies such as alterations in hormonal regulation, decreased insulin sensitivity, and impaired pancreatic function, all of which are associated with aging. Furthermore, the presence of a higher burden of chronic health conditions such as hypertension, cardiovascular disease, and metabolic disorders in the elderly may end in dysglycemia in sepsis states, as compared to the young (particularly less than 40 years of age). Another factor that can contribute to dysglycemia may be because of multiple medications, some of which may affect glycemic control. Age-related changes in appetite, metabolism, and nutritional intake can also impact glycemic control [14].

Here is a more detailed examination of several significant contributors: (1) decreased insulin sensitivity: with age, cells become less responsive to insulin, which is the hormone responsible for shuttling glucose from the bloodstream into cells. This decrease in insulin sensitivity, termed insulin resistance, paves the way for hyperglycemia [15]; (2) reduced beta-cell function: the pancreas houses beta cells, responsible for insulin production. Age-related decline in beta-cell function can lead to insufficient insulin production, further impacting blood sugar control [16]; and (3) impaired glucose counter-regulation: the body possesses mechanisms to prevent hypoglycemia. However, these mechanisms weaken with age, increasing the risk of hypoglycemia, particularly in older adults on insulin therapy [17].

Lifestyle choices also significantly influence dysglycemia risk at any age, but their impact becomes more pronounced with aging: (1) decreased physical activity: physical activity improves insulin sensitivity. As people age, physical activity levels often decline, further worsening insulin resistance and increasing dysglycemia risk [18]; (2) dietary changes: dietary patterns often shift with age. Older adults might consume more processed foods and saturated fats, both of which can contribute to insulin resistance and dysglycemia [19]; and (3) polypharmacy: many older adults take medications for various chronic conditions. Some medications can raise blood sugar levels, further complicating dysglycemia management [20].

Thirdly, we observed that the incidence of dysglycemia was higher in pneumonia and gastrointestinal (GI) sepsis, but it is not statistically significant. Both pneumonia and GI sepsis trigger a robust systemic inflammatory response characterized by the release of pro-inflammatory cytokines such as interleukin-6 (IL-6) and tumor necrosis factor-alpha (TNF-α). These cytokines contribute to insulin resistance and impair glucose uptake by peripheral tissues, leading to hyperglycemia. Critical illness, including pneumonia and GI sepsis, induces the release of stress hormones such as cortisol, catecholamines, and glucagon. These hormones mobilize glucose from hepatic glycogen stores through glycogenolysis and stimulate gluconeogenesis, leading to elevated blood glucose levels. Pneumonia and GI sepsis can disrupt the normal function of endocrine organs involved in glucose metabolism, affecting insulin secretion and exacerbating dysglycemia [10]. Further knowledge is not available in the literature on this aspect.

Fourthly, dysglycemia increased with an increase in APACHE II scores, as per our observations. The APACHE II scoring system incorporates several physiological parameters that reflect the degree of organ dysfunction. While APACHE II itself does not directly measure blood glucose levels, some of its components, such as the GCS score for neurological function, may indirectly indicate dysglycemia risk. For instance, hyperglycemia can worsen neurological status, potentially lowering the GCS score and contributing to a higher APACHE II score.

However, research on the ability of APACHE II to predict dysglycemia specifically remains inconclusive. Some studies suggest that a higher APACHE II score might be associated with a higher prevalence of hyperglycemia on admission [21]. However, others have not found a significant correlation [22]. This inconsistency highlights the complex interplay between various factors that contribute to dysglycemia in critically ill patients.

Dysglycemia as a risk factor in critical illness

Studies have consistently shown a strong association between dysglycemia and adverse outcomes in critically ill patients, including increased mortality, prolonged hospital stay, and higher rates of organ dysfunction [8].

Hyperglycemia is more commonly encountered and is thought to contribute to poor outcomes through various mechanisms. Elevated blood sugar levels can impair immune function, promote inflammation, and increase the risk of infections, all of which can worsen the underlying critical illness [23].

The statistically significant correlation observed between APACHE II scores and dysglycemia underscores the clinical relevance of using APACHE II as a prognostic tool in sepsis, as dysglycemia may serve as an indicator of disease severity. This can be due to many mechanisms such as higher APACHE II scores reflecting a more pronounced stress response to critical illness, characterized by increased release of stress hormones such as cortisol, catecholamines, and glucagon. These hormones can contribute to dysglycemia by promoting hepatic gluconeogenesis, glycogenolysis, and insulin resistance. The severity of illness captured by APACHE II scores reflects the extent of end-organ dysfunction, which can directly impact glucose metabolism, leading to dysglycemia. Patients with higher APACHE II scores are more likely to receive interventions such as vasopressors, corticosteroids, and sedatives, which can influence glycemic control. Vasopressors and corticosteroids, for instance, are known to increase blood glucose levels, while sedatives may potentiate hypoglycemia by blunting the counter-regulatory response to hypoglycemia [24].

## Conclusions

Dysglycemia is a frequent complication in non-diabetic patients with sepsis. It increased with age and APACHE II score, but it does not prolong the duration of hospital stay nor is it associated with the diagnosis. It is recommended to do further research to examine patient outcomes in terms of mortality/survival beyond 30 days. We also recommend conducting a multicenter blinded study following a similar approach.
